# Development of a Comprehensive Tool for School Health Policy Evaluation: The WellSAT WSCC

**DOI:** 10.1111/josh.12956

**Published:** 2020-11-12

**Authors:** Taylor A. Koriakin, Sarah L. McKee, Marlene B. Schwartz, Sandra M. Chafouleas

**Affiliations:** ^1^ Neag School of Education University of Connecticut, 249 Glenbrook Road, Unit 3064 Storrs CT 06269; ^2^ Rudd Center for Obesity and Food Policy University of Connecticut, 1 Constitution Plaza, Suite 600 Hartford CT 06103

**Keywords:** school health, whole child, policy evaluation, school health policy, WSCC model, WellSAT

## Abstract

**BACKGROUND:**

Stakeholders increasingly recognize the role of policy in implementing Whole School, Whole Community, Whole Child (WSCC) frameworks in schools; however, few tools are currently available to assess alignment between district policies and WSCC concepts. The purpose of this study was to expand the Wellness School Assessment Tool (WellSAT) for evaluation of policies related to all 10 domains of the WSCC model.

**METHODS:**

Developing the WellSAT WSCC was an iterative process that involved (1) identifying items for each domain based on key concepts and best practice recommendations; (2) expert review of the draft measure; (3) cognitive pre‐testing; (4) developing scoring criteria; and (5) pilot‐testing the measure.

**RESULTS:**

Ratings from expert reviewers indicated that the tool included items that were both relevant and important to each of the 10 WSCC domains. Results of cognitive pre‐testing indicated that the items were understood as intended. Feedback from expert reviews, cognitive pre‐testing, and pilot‐testing was used to further revise and refine the measure and develop a final version of the tool. Acceptable interrater reliability was established for the final selection of items.

**CONCLUSIONS:**

The WellSAT WSCC provides a reliable means for assessing integration and alignment between WSCC model concepts and district policies.

School health and wellness‐related fields are increasingly acknowledging the connections between student physical health, mental health, and academic outcomes.^1^, ^2^ Given these connections, the Whole School, Whole Community, Whole Child (WSCC) model^3^, ^4^ was developed to provide a framework for comprehensive whole child and coordinated school health approaches. The model depicts the child at the center and ensures that the whole child is supported and engaged across physical health, psychological health, academic, and social domains. The whole child is then surrounded by 10 domains impacting student wellbeing and outcomes. Rather than operating in isolation of each other, these 10 domains are depicted as working in tandem to support learning and health outcomes for the whole child through coordinating across policy, process, and practice. The whole model is then encompassed by the community environmental context surrounding the student.

## District Wellness Policies

Although WSCC initiatives may be implemented in policy, process, or practice, school leaders are increasingly emphasizing the importance of school building and district‐level policies to guide implementation of whole child and comprehensive school health initiatives.^5^, ^6^ District policies play an important role in employing the WSCC initiatives because they codify practices that schools are already implementing and facilitate consistent implementation over time. Policies are considered an important factor in the outer context of influences on school‐level adoption, implementation, and sustainment outcomes across a variety of domains.^7^ In addition, unlike other outer context factors such as community socioeconomic status or geographic location, policy is malleable^8^ by school leaders.

To date, the content of local wellness policies has been driven largely by federal legislation linked to child nutrition programs released in 2004^9^ and 2010.^10^ The regulations were written by the USDA with the primary goal of supporting nutrition and physical activity environments in schools that promotes student health. Specifically, these regulations require that all school districts participating in federal meal programs develop local wellness policies that address topics such as goals for nutrition and physical activity education; nutrition standards for school meals and competitive foods sold during the school day; and plans for evaluating the implementation of the wellness policy. Although there has been excellent compliance with the mandate to create wellness policies, 8 years of data from the National Wellness Policy Study document that these policies vary greatly with regard to both strength and comprehensiveness.^11^ For example, a study evaluating wellness policies in a nationally representative sample found that only 57% of schools surveyed in the 2014–2015 school year included all required wellness policy components as defined by federal law.^12^

State and regional studies have also found that the quality of wellness policies varies across districts. An early study that evaluated wellness policies for school districts in rural Colorado noted anecdotally that many contained “weak wording that produced minimal impact” (p. S141).^13^ In addition, Cox et al.^14^ conducted a systematic evaluation of wellness policies in states in the Southeastern United States and found that many policies were poorly written and thus difficult to enforce. Existing research further suggests that the quality and comprehensiveness of district wellness policies may vary by district characteristics, in particular the size of the school district.^14^, ^15^

## Wellness Policy Evaluation

To support research on the predictors, correlates, and consequences of wellness policies, researchers needed an objective, quantitative tool to measure policy strength and comprehensiveness. In 2009, a national team of researchers from Connecticut, Pennsylvania, Minnesota and Washington published an initial 96‐item measure to score wellness policies.^16^ Subsequently, this measure was shortened, updated with input from a stakeholder advisory group, placed online, and named the Wellness School Assessment Tool (WellSAT). The WellSAT was later updated to a 2.0 version^17^ in 2014 and a 3.0^18^ version in 2019 in order to accommodate changes in federal requirements and reflect current research and best practices. Each version of the measure has demonstrated interrater reliability.^16^, ^19^, ^20^ Data from registered users of the WellSAT website indicate that stakeholders from all 50 states have used the tool to code nearly 10,000 policies since the tool was published online.^20^

WellSAT 3.0 includes 67 items and evaluates wellness policies across 6 domains—Nutrition Education, Standards for USDA Nutrition Programs and School Meals, Nutrition Standards for Competitive Foods and Beverages, Physical Education and Physical Activity, Wellness Promotion and Marketing, and Implementation, Evaluation, and Communication. It is not an adequate tool, however, to assess school health as defined by the WSCC model because it primarily focuses on only 2 of the components—Nutrition Environment and Services and Physical Education and Physical Activity.

To our knowledge, there are 3 measures that have been updated or developed to support the assessment and study of the WSCC model. First, in 2017 the CDC updated and expanded its School Health Index (SHI)^21^ to allow schools to evaluate their practices related to WSCC implementation. However, the SHI focuses exclusively on practices; it does not evaluate the alignment between written district policies and the components of the WSCC model.

To fill this gap, 2 groups of researchers developed tools to study how written policies align with the full WSCC model. Chriqui et al^6^ evaluated the alignment between state‐level policies and the WSCC model across all 50 states. They noted that states varied greatly in terms of breadth and depth of coverage of WSCC domains in state‐level guidance and legislation. In addition, only 10 states had what they considered “deep” coverage of the WSCC model in their policies, defined as comprehensive and thorough policies related to at least 6 of 10 WSCC domains. Recently, this research team released another report that evaluated both local district‐level and state‐level policies in 20 states to evaluate how well they captured WSCC domains.^22^ Similar to their earlier findings,^6^ results indicated a great amount of variability between states and districts in terms of WSCC coverage in their policies. In addition, they found that state‐level policies were no more or less likely than district‐level policies to demonstrate greater breadth or depth of coverage. In addition, the coding scheme developed by this team was utilized to create a database on the National Association of State Boards of Education website^23^ which catalogs the overlap between state law and WSCC domains. In 2019, a second team of researchers published an evaluation of how Los Angeles County district policies align with the WSCC model.^24^ Across the 37 district policies reviewed, on average, only half of the items on the policy review measure were addressed. The authors also evaluated the strength of aligned policy language and found that fewer than 20% were strongly written.

The aim of the current study was to build upon this foundational work and develop an expanded version of the WellSAT that covers all of the domains of the WSCC model. We employed the same measure development process that was used with the most recent WellSAT 3.0 update, which included identifying best practices; input from a national group of advisors; cognitive interviewing; and usability and reliability testing.^19^ The objective was to create a public‐facing, user‐friendly measure that is widely accessible to a range of stakeholders, including school district administrators and members of district wellness committees.

## METHODS

### Measure Development

The process of developing the WSCC WellSAT items included 6 phases: (1) developing an initial set of items; (2) soliciting expert review related to the importance and relevance of each item; (3) conducting cognitive pre‐testing with experts; (4) developing scoring criteria; (5) pilot‐testing the measure; and (6) creating additional revisions to the measure and conducting further testing. Each phase is described below; development of the measure was an iterative process and revisions to the measure were made after each of the steps.

Developing items. Development of WellSAT WSCC items began with identifying key concepts from the WSCC model. We reviewed the CDC's descriptions of the 10 WSCC domains^4^ and identified key concepts outlined by the model within each domain. Each domain includes mention of concrete services to be provided, such as “psychological, psychoeducational, and psychosocial assessments,” in Counseling, Psychological, and Social Services and “first aid and emergency care” in Health Services. In addition, the model notes broader, more abstract concepts, such as “engaging families in a variety of meaningful ways” within the Family Engagement domain.

After identifying key concepts, the research team matched existing WellSAT 3.0 items to key concepts within each WSCC domain. Many existing WellSAT 3.0 items were retained to evaluate policies related to the Nutrition Environment and Services and Physical Activity and Physical Education domains; in addition, some items were adapted for use in the Family Engagement, Community Involvement, and Employee Wellness domains. In Table 1, items mirroring those on the most updated version of the WellSAT 3.0 are depicted in italics. Readers should note that all WellSAT 3.0 items included in the WellSAT WSCC measure were validated through a separate process;^20^ therefore, this study included development and evaluation of *only* items that were unique to the WellSAT WSCC. In Table 2, an overview of the overlap between WellSAT WSCC and WellSAT 3.0 items is provided.

**Table 1:** Final WellSAT WSCC Measure

Domain	Items
Physical Education and Physical Activity (PEPA)	PEPA1. There is a written physical education curriculum for grades K‐12 PEPA2. The written physical education curriculum for each grade is aligned with national and/or state physical education standards. PEPA3a. Addresses time per week of physical education instruction for all elementary school students. Use N/A if no elementary school in district. PEPA3b. Addresses time per week of physical education instruction for all middle school students. Use N/A if no middle school in district. PEPA3c. Addresses time per week of physical education instruction for all high school students. Use N/A if no high school in district. PEPA4. Addresses qualifications for physical education teachers for grades K‐12 PEPA5. Addresses before and after school physical activity for all students including clubs, intramural, interscholastic opportunities PEPA6. Addresses recess for all elementary school students. Use N/A if no elementary schools in district PEPA7. Addresses physical activity breaks during school. PEPA8. Addresses physical activity not being used as a punishment. PEPA9. Addresses physical activity not being withheld as a punishment.
Nutrition Environment and Services (NES)	NES1. Assures compliance with USDA nutrition standards for reimbursable school meals. NES2. Addresses compliance with USDA nutrition standards (commonly referred to as Smart Snacks) for all food and beverages sold to students during the school day. NES3. Addresses fundraising with food to be consumed during the school day. NES4. Free drinking water is available during meals. NES5. Addresses availability of free drinking water throughout the school day. NES6. District takes steps to protect the privacy of students who qualify for free or reduced priced meals. NES7. Addresses how to handle feeding children with unpaid meal balances without stigmatizing them. NES8. Specifies strategies to increase participation in school meal programs. NES9. Links nutrition education with the food environment. NES10. Addresses the amount of “seat time” students have to eat school meals. NES11. Addresses purchasing local foods for the school meals program. NES12. Specifies marketing to promote healthy food and beverage choices. NES13. Restricts marketing on the school campus during the school day to only those foods and beverages that meet Smart Snacks standards. NES14. Addresses food not being used as a reward. NES15. Regulates food and beverages served at class parties and other school celebrations in elementary schools.
Health Education (HE)	HE1. Addresses health education for students in district (eg, hours, semesters, etc.). HE2. Specifies that health education is provided by qualified, trained professionals. HE3. Includes topics for health education that are designed to promote student wellness. HE4. Includes goals for nutrition education that are designed to promote student wellness. HE5. Addresses alignment between health education curriculum goals and the needs of students in the community. HE6. Addresses opportunities for interdisciplinary connections and practicing health‐related skills outside of health education classes. HE7. Addresses National Health Education Standards (NHES). HE8. Incorporates the CDC's characteristics of an effective health education curriculum. HE9. Specifies that health education curriculum will be evaluated and revised.
Social & Emotional Climate (SEC)	SEC1. Addresses participation in school climate surveys. SEC2. Addresses sharing aggregate results of school climate data with stakeholders. SEC3. Addresses promoting positive relationships between students and employees. SEC4. Identifies school‐wide approaches to address harassment, bullying, and/or cyberbullying. SEC5. Addresses diversity and inclusion to promote engagement of all students in school activities. SEC6. Addresses reviewing and responding to school climate data. SEC7. Addresses use of positive behavior support practices. SEC8. Addresses minimization of exclusionary disciplinary practices (eg, suspension and expulsion).
Safe Environment (SE)	SE1. Identifies regular cleaning and maintenance practices for district buildings. SE2. Addresses prevention and safe removal (if applicable) of mold and moisture in district buildings. SE3. Addresses reduction/minimization of student and staff exposure to toxins (eg, vehicle exhaust, mold, air pollution, pesticides, cleaning products). SE4. Addresses air quality and ventilation for district buildings and grounds. SE5. Specifies system for monitoring and addressing water quality in district buildings. SE6. Specifies an integrated pest management plan. SE7. Addresses district buildings' physical condition including lighting, noise, ventilation, moisture, and temperature during normal operating hours and construction.
	SE8. Addresses student and employee involvement in maintaining the school physical environment. SE9. Addresses maintenance of facilities and compliance to safety standards. SE10. Specifies physical safety measures (eg, double entry access, surveillance, locked doors and windows) and/or procedures in district buildings and grounds (eg, active supervision of hallways, check in check out systems for visitors, safe transport). SE11. Addresses the establishment on an ongoing school safety team. SE12. Specifies a crisis preparedness and response plan. SE13. Addresses presence of and training for school resource officers in district buildings (if applicable).
Health Services (HS)	HS1. Addresses presence of qualified health service providers in district schools. HS2. Addresses communication and care coordination with community‐based healthcare providers to meet student health needs. HS3. Addresses school health service provider consultation and collaboration with other school staff to respond to a broad range of student health needs. HS4. Addresses alignment of health services with the health needs of students in the community. HS5. Addresses engagement of and communication with families to address individual student health needs. HS6. Specifies opportunities for dissemination of health information resources to students and families (eg, pamphlets, flyers, posters). HS7. Addresses student physical health screenings (eg, vision, hearing). HS8. Addresses assessment and planning for chronic disease management to meet individual student needs (eg, asthma, diabetes, etc.) HS9. Addresses management of allergies in the school environment. HS10. Addresses provision of acute and emergency care. HS11. Specifies a health services response to student sexual risk behavior (HIV/STD). HS12. Specifies a health services response to student substance use (eg, opioid overdose prevention policy).
Behavioral Supports (BS)	BS1. Addresses methods and procedures to identify students with social, emotional, and/or behavioral (SEB) needs (ie, what methods or procedures are in place if a student has a suspected behavioral risk). BS2. Identifies an internal (within school) referral systems for SEB needs (eg, Student Assistant or Student Support Team or other internal referral system or other means by which the student will gain access to service after identification). BS3. Addresses presence of credentialed behavioral health service providers in district schools (eg, social workers, school psychologists, and/or school counselors). BS4. Addresses use of evidence‐based prevention and intervention strategies to meet a continuum of SEB needs. BS5. Defines a data‐driven process for monitoring response to supports for students with SEB needs. BS6. Addresses communication and care coordination with community‐based providers to meet student SEB needs. BS7. Addresses engagement of and communication with families to address SEB needs.
Employee Wellness (EW)	EW1. Designates employee wellness as a priority in the district organization structure. EW2. Addresses sharing of health education materials with school employees. EW3. Addresses coordination with health insurance providers to conduct health risk screening. EW4. Addresses creating an environment that supports employees' healthy lifestyles. EW5. Addresses social and emotional supports for school employees including the use of Employee Assistance Programs or other programs. EW6. Includes use of employee input in design and evaluation of employee wellness programs. EW7. Addresses tobacco use by school employees. EW8. Encourages staff to model healthy eating and physical activity behaviors. EW9. Addresses promotion of a positive workplace climate. EW10. Addresses space and break time for lactation/breast feeding. EW11. Addresses methods to encourage participation in available wellness programs.
Community Involvement (CI)	CI1. Addresses community representation on district wellness committee. CI2. Addresses how community stakeholders will participate in the development, implementation, and periodic review and update of the local wellness policy. CI3. Addresses making the wellness policy available to the public. CI4. Joint or shared‐use agreements for physical activity participation at all schools. CI5. Specifies community‐based opportunities for student service learning.
Family Engagement (FE)	FE1. Addresses family representation on district wellness committee. FE2. Addresses how families will participate in the development, implementation, and periodic review and update of the local wellness policy. FE3. Addresses opportunities for ongoing, sustained family engagement throughout the school year. FE4. Addresses regular 2‐way communication with families. FE5. Addresses alignment of family engagement activities and the needs of the community. FE6. Addresses alignment of family engagement programs and district wellness objectives. FE7. Addresses use of culturally responsive practices to engage families. FE8. Addresses sharing wellness‐related information with families. FE9. Addresses school‐based volunteer opportunities for families (eg, parent teacher associations, parent teacher organizations, family‐school committees).
Implementation, Integration, and Evaluation (IIE)	IIE1. Specifies use of Centers for Disease Control and Prevention's WSCC model or other coordinated/comprehensive method to guide wellness activities. IIE2. Addresses the establishment of an ongoing district wellness committee. IIE3. Addresses how all relevant stakeholders (parents, students, representatives of the school food authority, teachers of physical education, school health professionals, the school board, school administrator, and the general public) will participate in the development, implementation, and periodic review and update of the local wellness policy. IIE4. Addresses diverse representation on district wellness committee outside of federal requirements to reflect WSCC domains (eg, behavioral health, physical environment, employee wellness). IIE5. Addresses the establishment of an ongoing school building level wellness committee. This may also be called a school health team, school health advisory committee, or similar name. IIE6. Identifies the officials responsible for implementation and compliance with the wellness policy. IIE7. Addresses the assessment of district implementation of the local wellness policy at least once every 3 years. IIE8. Addresses a plan to assess the impact of wellness policy on behavioral health and educational outcomes, including a person/group responsible for tracking outcomes (eg, student and employee attendance, office discipline referrals, BMI screenings). IIE9. Triennial assessment results will be made available to the public and will include: 1. The extent to which schools under the jurisdiction of the LEA are in compliance with the local school wellness policy; 2. The extent to which the LEA's local school wellness policy compares to model local school wellness policies; 3. A description of the progress made in attaining the goals of the local school wellness policy. IIE10. Addresses a plan for updating policy based on results of the triennial assessment. IIE11. Addresses use of culturally inclusive practices in school wellness activities. IIE12. Identifies funding support for wellness activities. IIE13. Identifies professional learning opportunities for district employees to support wellness policy implementation.

Items written in italics are items that are also included in the WellSAT version 3.0.

**Table 2:** WellSAT 3.0 and WellSAT WSCC Alignment

Domain	Total WellSAT WSCC Items	WellSAT 3.0 Items	Items Unique to WellSAT WSCC
Behavioral Supports	7	0	7
Community Involvement	5	2	3
Employee Wellness	11	1	10
Family Engagement	9	0	9
Health Education	9	1	8
Health Services	11	0	11
Nutrition Environment	15	15	0
Physical Activity and Physical Education	9	9	0
Safe Environment	13	0	13
Social and Emotional Climate	10	0	10
Implementation, Integration, and Evaluation	13	7	6
Full Scale	112	35	77

To develop new items for domains that the WellSAT 3.0 does not address, the research team consulted with policy recommendations from national organizations aligned with each domain. For example, for the Counseling, Psychological, and Social Services (renamed as “Behavioral Supports” in our measure), we consulted practice models and policy guidance from the National Association for School Psychologists, School Social Work Association of America, and the American School Counselor Association. We then cross referenced these policy recommendations with the list of key concepts outlined by the WSCC model for each domain. The final list of WellSAT WSCC items, each item's overlap with the model, and related policy recommendations are available in Appendix A. In addition to developing items for each of the 10 domains, we also identified key concepts to address the integration and coordination of WSCC‐related policies across domains. Given that a primary goal of the model is the coordination of supports across each domain, we created an additional domain focused on the higher‐level coordination of each of the elements of the model. This 11th domain was titled the “Integration, Implementation, and Evaluation (IIE).”

We next created a set of preliminary items and a draft version of the measure. This initial version of the measure was arranged into 11 sections (one for each WSCC domain, plus the IIE domain) with approximately 10 items per section. This mirrors the structure of the WellSAT 3.0,^20^ which has sections grouped by the focus of the items, and a mean of 10.7 items per section.

Expert review process. After developing an initial set of items, the research team conducted an expert review process regarding the preliminary items on the measure. We identified 2 levels of review: (1) those with experience in specific content areas to provide feedback on a single domain of the measure and (2) and reviewers to provide feedback for the entire measure. Reviewers included researchers with expertise in school wellness or other WSCC domains, employees of departments of public health and education, administrators of large public‐school districts, and leaders of national non‐profit advocacy organizations. In March and April of 2018, experts were invited to provide feedback via a Qualtrics survey; a total of 24 experts participated in the feedback and review process.

Reviewers were asked to provide both quantitative and qualitative feedback for items. First, expert reviewers were instructed to rate each item on importance and relevance. Relevance was defined for participants as the degree to which the item is closely connected or appropriate to policy in the respective domain (1 = low/not relevant, 2 = somewhat relevant, 3 = highly relevant); whereas importance was defined as the degree to which is each item is of significance or provides value in school policy assessment (1 = low importance, 2 = somewhat important, 3 = highly important). For qualitative feedback, reviewers were given the opportunity to provide comments and feedback for individual items and about the entire domain such as if they felt any constructs were missing and if the items captured the domain as a whole.

Cognitive pre‐testing. We performed cognitive pretesting^25^ on our final item selections with 2 school policy experts from state departments of education. In each pretest, the respondent was asked to read each question aloud, then discuss how he or she would respond to the item, noting any confusion or comments that arose. For some items, the interviewer prompted the respondent to elaborate, using phrases such as “In your own words, what is this question asking?” or inquiring about the respondent's understanding of specific phrases. A final probe followed each of the sections to gauge whether the section captured the entire domain as a whole, if anything was missing, and if there were any items the respondent did not believe would be included in a policy.

### Scoring Criteria

After selecting all of the items to be included in the draft measure, scoring criteria were developed to objectively evaluate policy language related to each item. An example of item‐specific scoring guidelines is provided in Table 3 using an item from the Social and Emotional Climate domain. Policies that do not address the topic area targeted by an item receive a rating of 0. Policies that address the topic, but are weakened by vague language, loopholes, or statements that are written as aspirations or recommendations receive a rating of 1. Policies that address the topic and use clear, specific language that requires action or regulation receive a rating of 2.

**Table 3:** Scoring Guidelines for WellSAT WSCC Items

Score	General Guidelines	Guidelines for SEC4
0	Not mentioned in policy language; no reference to relevant laws	Not mentioned
1	Recommended	Recommends that schools develop approaches for preventing and responding to bullying and harassment
	Example language includes: may, should, can, could, “The District seeks to…”, “The district aspires to…”	
2	Mandated	Requires that schools develop approaches for preventing and responding to bullying and harassment
	Example language includes: shall, will, “It is the responsibility of the [role] to…”, required, must	

### Pilot‐Testing

The full draft measure was pilot‐tested on a sample of district policies. Thirty Connecticut school districts were selected for scoring in our study. Connecticut's 161 school districts are grouped into 9 District Reference Groups (DRGs) to allow for more meaningful comparisons between socioeconomically similar districts. We used a random number generator to select 3 districts from each DRG plus 3 additional districts from the 3 DRGs with the greatest number of districts for a total of 30.

Policy collection. Each district's policies (including Board or Education policies, any superintendent regulations, and any administrative guidelines) were obtained via the district's website. When policies cited state or federal law related to measure items, we scored the text of the cited law according to the same scoring criteria used for policy statements. Districts could receive a higher score than the standardized coding for laws if they included language that was stronger than the federal or state law.

Scoring. Six research assistants participated in coding district policies. Two graduate research assistants who participated in development of the items and scoring guidelines served as leaders, training the other 4 coders. The research team conducted an initial 2‐hour training to introduce the coders to the measure, items, and scoring guidelines. Following the initial training, all coders scored the same 3 districts' policies and met to discuss discrepancies after each district was completed. The remaining 27 districts' policies were assigned individually to one of the 6 research assistants, with each individual coding 4 to 6 district policies. The coding team met periodically during this time to address questions that arose during scoring and to reach a consensus on policies for which scoring was unclear.

Interrater reliability. After the initial coding was complete, steps were taken to conduct analyses to confirm adequate interrater reliability. Of the 30 districts 6 (20% of districts coded) were randomly selected for double coding. These districts were then independently re‐coded by a group of 3 research assistants. Interrater reliability was calculated by percent agreement; each item was coded as 1 if the item was coded was an exact match between the 2 raters (eg, both coded the item as 2) or 0 if there was a discrepancy between the 2 raters (eg, one rater coded the item as “0” and the other coded it as 2).

### Additional Revisions and Further Testing

After conducting the initial round of pilot‐testing, revisions were made to the measure based on feedback from the coders on common areas of confusion and to clarify wording that was unclear. In addition, further detail and examples were provided to assist in accurate coding for items. In particular domains with the lowest interrater reliability for the initial pilot‐testing were reviewed carefully to provide further detail and guidance for these items. After these revisions, another 6 Connecticut districts were selected for coding using the most updated version of the measure. Two research assistants were assigned to code 3 districts each and a third research assistant double‐coded all 6 districts. Interrater reliability was again calculated to evaluate if revisions to the measure facilitated improved standardized and accurate coding.

## RESULTS

### Expert Review

A summary of ratings from the expert review process is provided in Table 4. The table provides the total number of reviewers of that domain (including those that reviewed only that domain of items and those that reviewed that domain along with the entire measure) and the average ratings for importance and relevance across reviewers. Although there was some variability between domains, overall the averages across domains indicated that items selected for the measure were interpreted as important and relevant to constructs outlined in the WSCC model.

**Table 4:** Mean Expert Review Ratings for Preliminary Scale by Domain

Domain	Number of Reviewers	Number of Items	Importance M (SD)	Relevance M (SD)
Behavioral Supports	6	10	2.86 (0.19)	2.88 (0.15)
Community Involvement	5	7	2.74 (0.33)	2.68 (0.19)
Employee Wellness	5	10	2.88 (0.2)	2.80 (0.13)
Family Engagement	5	8	2.71 (0.25)	2.56 (0.27)
Health Education	6	10	2.69 (0.21)	2.75 (0.26)
Health Services	5	12	2.74 (0.25)	2.74 (0.25)
Nutrition Environment	7	14	2.96 (0.35)	2.77 (0.08)
Physical Environment	6	13	2.74 (0.25)	2.70 (0.23)
Physical Activity and Physical Education	6	9	2.82 (0.29)	2.76 (0.28)
Social and Emotional Climate	6	10	2.92 (0.11)	2.92 (0.13)
Implementation, Integration, and Evaluation	4	11	2.93 (0.17)	2.86 (0.09)

Additional revisions, including adding and removing items, were made after expert review was conducted; therefore, the number of items in each domain presented in this table does not necessarily correspond exactly with the number of items on each domain in the final measure presented in Table 1.

### Cognitive Pre‐Testing

In general, respondents understood most items appropriately. We found that the most substantial potential barriers to appropriate use of the WellSAT WSCC were misunderstandings regarding which documents are to be evaluated by coders. In addition, given the comprehensiveness of the tool, reviewers shared that it may be difficult and cumbersome to review all of a district's policies at the same time. To provide additional assistance to potential users, we provided guidelines for suggested policies to review to complete the items in each domain (eg, for the Health Education domain coders were instructed to review curriculum and instruction, sexual health education, and substance use prevention policies). These suggested policies to review are also included in the full version of the final measure to assist in locating relevant policy language. In addition, we used this feedback to clarify wording and shape the scoring guidelines of several items, to rearrange the order of items within sections, and to develop instructions for use of the WellSAT WSCC through the creation of a user guide.

### Interrater Reliability

Results of interrater reliability results are presented in Table 5. For the initial round of pilot‐testing, interrater reliability was 76.17% for the full scale, with individual domains ranging between 61.9% (Behavioral Supports) to 97.51% (Physical Environment). As noted above, we sought to revise and provide further clarification for items on domains with the lowest interrater reliability, in particular those below 70% reliability (Behavioral Supports, Community Involvement, and Health Services). After making additional revisions and conducting another smaller round of testing, we again evaluated interrater reliability (noted as Round 2 in Table 5). For Round 2, the interrater reliability for the whole scale improved to 85.49%; for individual domains, interrater reliability ranged from 75.93% (Family Engagement) to 88.89% (IIE). In addition, interrater reliability was at or above 75% for all domains.

**Table 5:** Inter‐rater Reliability by Domain

Domain	Round 1 (%)	Round 2 (%)
Behavioral Supports	61.90	88.09
Community Involvement	66.68	83.33
Employee Wellness	76.67	86.68
Family Engagement	79.17	75.93
Health Education	83.33	84.23
Health Services	65.28	86.37
Physical Environment	87.51	87.61
Social and Emotional Climate	81.67	83.33
Implementation, Integration, and Evaluation	83.33	88.89
Full Scale	76.17	85.49

### Final Measure

After incorporating feedback throughout all steps of development, the final measure included 112 items (see Table 1). As previously noted, the overlap between WellSAT 3.0 and WellSAT WSCC is depicted in Table 2. Some domains such as Nutrition Environment and Physical Education and Activity are completely derived from WellSAT 3.0 items, whereas others such as Behavioral Supports and Social & Emotional Climate have no overlap with WellSAT 3.0 items. Other domains such as Community Involvement and IIE are comprised of a combination of items from WellSAT 3.0 and new items created for this new measure.

## DISCUSSION

The purpose of this study was to expand the existing WellSAT 3.0 measure to encompass all 10 domains of the WSCC model represented in district wellness policies. We utilized a multi‐step, iterative process to develop the measure including expert review, cognitive pre‐testing, and pilot‐testing. This process resulted in a final version of the WellSAT WSCC measure comprised of 112 items across the 10 domains of the WSCC model and an additional domain to evaluate implementation, integration, and evaluation of supports across domains.

Feedback from expert review indicated that the selected items captured relevant and important constructs related to each domain, and cognitive pre‐testing participants reported that most items were correctly understood and interpreted. Results of the initial round of pilot‐testing indicated generally acceptable levels of interrater reliability, with feedback from coders and analysis of patterns of interrater reliability used to make further revisions to items and scoring criteria to ultimately result in improved reliability. Reliability estimates for the WellSAT WSCC measure were similar to those reported for the original WellSAT measure.^16^

Although this study resulted in an overall measure with adequate reliability and construct validity, it should be noted that the individual domains were not equal in terms of ease of development. The WSCC model varies between domains in terms of the detail of both the description of the domain and specificity of suggested best practices in that area. In line with this, there are also differences between domains in terms of the research support for best practices and how readily policy guidance related to that area is available from national organizations. For example, while some domains are associated with strong empirical support and specific policy recommendations, other domains like Family Engagement and Community Involvement had much less concrete guidance available. Therefore, this represented an initial challenge to develop unique items for these areas. Developing the entire measure was an iterative process with changes made after each step in the process; however, there more iteration was required for some domains than others. Some domains with more limited guidance were also associated with greater variability in expert review ratings and were more difficult to establish adequate reliability.

The WellSAT WSCC offers a WSCC‐aligned policy evaluation tool that is unique in several ways. First, our tool capitalizes on the familiarity of the format and style of the existing WellSAT measure to deliver a public‐facing tool school districts and other stakeholders can use to evaluate policy strength and comprehensiveness. Second, we used an iterative process to develop the measure and sought feedback from intended users at multiple points in the development process to ensure that the measure reflects current research and best practices. We also engaged a national panel of content experts, including other researchers who have conducted foundational work in evaluating WSCC‐related policies.^6^, ^22^, ^24^ Third, we conducted interrater reliability testing with several coders to ensure that the measure can be used reliably by a range of users. Finally, we created a user manual, videos, and other scoring support materials which are all hosted on the University of Connecticut's Collaboratory for School and Child Health (https://csch.uconn.edu/wellsat‐wscc/) and Rudd Center for Food Policy and Obesity (http://uconnruddcenter.org/wellsat‐wscc) websites for public use.

### Limitations

The WellSAT WSCC represents a new and unique measure; thus, there are associated limitations. First, our initial testing of the measure was limited to Connecticut school districts which may not reflect the landscape of district policies nationwide. However, in our expert review process, we solicited feedback for those with expertise in school wellness outside of Connecticut to ensure a broad range of perspectives across domains pertaining to school wellness. Second, given that the WellSAT WSCC is a new measure, this is the first study to provide psychometric evidence of how the tool functions. Additional evaluation of the tool will be needed to not only see how it functions in evaluating district policies outside of Connecticut but also if the measure is consistent and valid compared to other policy evaluation measures. Additional research should also seek to determine if use of the tool is associated with improved implementation of WSCC supports and thus student and staff outcomes.

### Conclusions

In sum, written policy can be a powerful tool in promoting WSCC school health initiatives. As one of our cognitive pre‐testing reviewers remarked, “If you don't write it down, it isn't anything.” This study sought to create a new policy evaluation tool, expanding from the strengths of the existing WellSAT 3.0 to encompass the WSCC model domains. Results indicated that the WellSAT WSCC captured relevant and important constructs related to each domain, and adequate interrater reliability was achieved through initial pilot‐testing of the measure. This publicly accessible measure is accompanied by supportive tools and guides to facilitate the policy review process for a range of users who are dedicated to supporting the whole child and improving school wellness through use of the WSCC model.

## IMPLICATIONS FOR SCHOOL HEALTH

The WellSAT WSCC provides school districts with a freely accessible tool to evaluate alignment between their policies and the WSCC model. In addition, the WellSAT WSCC website (https://csch.uconn.edu/wellsat‐wscc/) provides additional resources to aid stakeholders in policy evaluation including instructional videos, a user guide, and action planning tools. Anecdotally, we have received feedback that although school districts are interested in engaging in policy review, they often times are not sure where to start in their evaluation. We provide the following recommendations for getting started with policy evaluation and using the WellSAT WSCC tool:

- If the district is overwhelmed by reviewing policies related to all domains, we recommend that the team consider prioritizing 2 to 3 domains for policy review. Specific guidance on how to prioritize can be found in the action planning tools located in the appendices of the user manual on the WellSAT WSCC website . In addition, stakeholders may select domains that are aligned with strategic goals or other district‐wide initiatives.
- First, the team should gather district employees with expertise related to the areas of policy review. In addition to wellness committee members, it may also be appropriate to include those district and school personnel with roles and/or responsibilities related to WSCC domains in the policy evaluation process. For example, if Social & Emotional Climate is selected for review, it may be relevant to include members of school climate and culture teams and school mental health professionals to be part of the review.
- Then, the team should download and review the 3 guiding documents from the WellSAT WSCC website: (1) the User Manual, which provides an overview of the measure and answers frequently asked questions; (2) the Coding Guide, which includes detailed scoring criteria for each item; and (3) the Score Sheet for documenting scores for each item.
- The policy review team should then locate district‐level policy documents related to the selected domains for review. These are typically located on the local Board of Education page on districts' websites. We recommend reviewing all Board of Education policies, administrative regulations, and federal and state law related to the domain of interest. The WellSAT WSCC User Manual also provides suggestions of policies to review for each domain.
- Then, the team can begin reviewing policy documents and coding items for selected domains. The WellSAT WSCC Coding Guide provides detailed guidance for how to score each item.
- As the team is coding, members can use the WellSAT WSCC Score Sheet to document scores. Once the review is complete, strength and comprehensiveness scores should be calculated for each domain.
- Finally, the policy review team should use the results to create an action plan with the district wellness committee. If a domain received a low score, the team should consider what steps can be taken to strengthen policy language or better implement existing policies in this domain.

### Human Subjects Approval Statement

This study evaluated publicly available policy documents and as such was exempt from human subjects review.

### Conflict of Interest

All authors of this article declare they have no conflicts of interest.

## WellSAT WSCC Alignment and Item Sources

**Table:**

WellSAT WSCC Item	CDC WSCC Model text	Source
Behavioral Supports		
BS1. Addresses methods and procedures to identify students with social, emotional, and/or behavioral (SEB) needs	“…systems‐level assessment, prevention, intervention, and program design by school‐employed mental health professionals contribute to the mental and behavioral health of students as well as to the health of the school environment.”	WSCC description^26^
BS2. Identifies internal (within school) referral systems to address SEB needs	“Services include…referrals to school and community support services as needed”	National Association of School Psychologists (NASP) Practice Model,^27^ Social Work Practice Model,^28^ WSCC description^26^
BS3. Addresses presence of credentialed behavioral health service providers appropriate for student population needs (eg, social workers, school psychologists, and/or school counselors)	“Professionals such as certified school counselors, school psychologists, and school social workers provide these services.”	WSCC description^26^
BS4. Addresses use of evidence‐based prevention and intervention strategies to meet a continuum of SEB needs	“Services include…direct and indirect interventions to address psychological, academic, and social barriers to learning, such as individual or group counseling and consultation…”	WSCC description^26^
BS5. Defines a data‐driven process for monitoring response to supports for students with SEB needs	See BS1	NASP Practice Model,^27^ Social Work Practice Model,^28^ ASCA National Model^29^
BS6. Addresses community‐based service coordination and communication with providers to meet student SEB needs	“School employed mental health professionals ensure that services provided in school reinforce learning and help to align interventions provided by community providers within the school environment”	WSCC description^26^
BS7. Addresses engagement of and communication with families to address SEB needs	“…school‐community‐family collaboration”	WSCC description^26^
Social Emotional Climate		
SEC1. Addresses participation in school climate surveys	“A positive social and emotional school climate is conducive to effective teaching and learning.”	Center for Social Emotional Education,^30^ National School Climate Standards^31^
SEC2. Addresses sharing aggregate results of school climate data with stakeholders	See SEC1	National School Climate Standards^31^
SEC3. Addresses promoting positive relationships between students and employees	“The social and emotional climate of a school can impact… relationships with other students, staff, family, and community…”	National School Climate Standards^31^
SEC4. Identifies school‐wide approaches to address harassment, bullying, and/or cyberbullying	“Such climates promote health, growth, and development by providing a safe and supportive learning environment.”	NASP Practice Model^27^
SEC5. Addresses diversity and inclusion to promote engagement of all students in school activities	“The social and emotional climate of a school can impact student engagement in school activities…”	Center for Social Emotional Education,^30^ National School Climate Standards^31^
SEC6. Addresses reviewing and responding to school climate data	See SEC1	Center for Social Emotional Education^30^
SEC7. Addresses use of positive behavior support practices	“Social and Emotional School Climate refers to the psychosocial aspects of students' educational experience that influence their social and emotional development.”	Center for Social Emotional Education^30^
SEC8. Addresses minimization of exclusionary disciplinary practices (eg, suspension and expulsion)	“The social and emotional climate of a school can impact student engagement in school activities; relationships with other students, staff, family, and community; and academic performance.”	NASP Practice Model^27^
SEC9. Addresses social emotional learning (SEL)	See SEC7	Collaborative for Academic, Social, and Emotional Learning (CASEL) Key Features of High‐Quality Policies^32^
SEC10. Connects social emotional learning standards and academic standards	“The social and emotional climate of a school can impact…academic performance.	CASEL Key Features of High‐Quality Policies^32^
Safe Environment		
SE1. Identifies regular cleaning practices for district buildings	“A healthy school environment will address a school's physical condition during normal operation as well as during renovation (eg, ventilation, moisture, temperature, noise, and natural and artificial lighting), and protect occupants from… biological and chemical agents in the air, water, or soil as well as those purposefully brought into the school (eg, pollution, mold, hazardous materials, pesticides, and cleaning agents).”	United States Environmental Protection Agency (EPA)^33^
SE2. Addresses prevention and safe removal (if applicable) of mold and moisture in district buildings	See SE1	EPA^33^
SE3. Addresses minimization of student and staff exposure to toxins (eg, vehicle exhaust, mold, air pollution, pesticides, cleaning products)	See SE1	EPA^33^
SE4. Specifies a system for monitoring and addressing air quality and ventilation for district buildings and grounds	See SE1	EPA^33^
SE5. Specifies system for monitoring and addressing water quality in district buildings	See SE1	EPA^33^
SE6. Specifies an integrated pest management plan	See SE1	EPA^33^
SE7. Addresses district buildings' physical condition such as lighting, noise, and temperature during normal operating hours and construction	See SE1	WSCC description^26^
SE8. Addresses student and employee involvement in maintaining the school physical environment (eg, graffiti, littering, recycling)	See SE1	EPA^33^
SE9. Addresses maintenance of facilities and equipment and compliance to safety standards	“A healthy school environment will … protect occupants from physical threats (crime, traffic, injuries)”	EPA^33^
SE10. Specifies physical safety measures (eg, double entry access, surveillance, locked doors and windows) and/or procedures in district buildings and grounds (eg, active supervision of hallways, check in check out systems for visitors, safe transport)	See SE9	NASP School Safety Policy Recommendations, Prepare Model^34^, ^35^
SE11. Addresses the establishment of an ongoing school safety team	See SE9	NASP School Safety Policy Recommendations, Prepare Model^34^, ^35^
SE12. Specifies a crisis preparedness and response plan	See SE9	NASP School Safety Policy Recommendations, Prepare Model^34^, ^35^
SE13. Addresses training for school resource officers in district buildings (if applicable)	See SE9	NASP School Safety Policy Recommendations, Prepare Model ^34^, ^35^
Community Involvement		
CI1. Addresses community representation on district wellness committee	“The school, its students, and their families benefit when leaders and staff at the district or school solicits and coordinates information, resources, and services available from community‐based organizations, businesses, cultural and civic organizations, social service agencies, faith‐based organizations, health clinics, colleges and universities, and other community groups.”	Derived from WellSAT 3.0 item IEC2;^11^ Healthy and Hunger‐Free Kids Act (HHFKA) ^10^
CI2. Addresses community stakeholders participation in the development, implementation, and periodic review and update of the local wellness policy	See CI2	Derived from WellSAT 3.0 item IEC2;^20^ HHFKA^10^
CI3. Addresses shared‐use agreements between school and community	“Schools, students, and their families can contribute to the community… by sharing school facilities with community members (eg, school‐based community health centers and fitness facilities”	WellSAT 3.0 item PEPA15^20^
CI4. Specifies community‐based opportunities for student service learning	“Schools, students, and their families can contribute to the community through service‐learning opportunities…”	WSCC description^26^, ^36^
CI5. Addresses availability of the wellness policy to the public (Federal Requirement: the LEA must make the current policy available to the public on an annual basis)	“Community groups, organizations, and local businesses create partnerships with schools, share resources, and volunteer to support student learning, development, and health‐related activities”	WellSAT 3.0 item IEC4;^20^ HHFKA^10^
Family Engagement		
FE1. Address family representation on district wellness committee	“Families and school staff work together to support and improve the learning, development, and health of students.”	Derived from WellSAT 3.0 item IEC2;^20^ HHFKA^10^
FE2. Addresses family participation in the development, implementation, and periodic review and update of the local wellness policy	See FE1	Derived from WellSAT 3.0 item IEC2;^20^ HHFKA^10^
FE3. Addresses providing opportunities for ongoing, sustained family engagement throughout the school year	“School staff are committed to…engaging families in a variety of meaningful ways…” “School staff are committed to… sustaining family engagement”	WellSAT 3.0 item PEPA11;^20^ WSCC description^26^
FE4. Addresses regular, 2‐way communication with families	“Family engagement with schools is a shared responsibility of both school staff and families.”	Family engagement policy guidance^37^, ^38^, ^39^
FE5. Addresses alignment of family engagement activities with the needs of the community	See FE3	Family engagement policy guidance^37^, ^38^, ^39^
FE6. Addresses alignment of family engagement programs and district wellness objectives	“This relationship between school staff and families cuts across and reinforces student health and learning in multiple settings—at home, in school, in out‐of‐school programs, and in the community.”	Family engagement policy guidance^37^, ^38^, ^39^
FE7. Addresses use of culturally responsive practices to engage families	See FE3	Family engagement policy guidance^37^, ^38^, ^39^
FE8. Addresses sharing wellness‐related information with families	“This relationship between school staff and families cuts across and reinforces student health and learning in multiple settings—at home, in school, in out‐of‐school programs, and in the community.”	Family engagement policy guidance^37^, ^38^, ^39^
FE9. Recommends that school‐based volunteer opportunities be provided for families (eg, parent teacher associations, parent teacher organizations, family‐school committees)	“Families are committed to actively supporting their child's learning and development.”	Family engagement policy guidance^37^, ^38^, ^39^
Health Education		
HE1. Includes topics for health education that are designed to promote student wellness in a manner that the local education agency determines is appropriate and aligned with state requirements	“Health education helps students acquire the knowledge, attitudes, and skills they need for making health‐promoting decisions, achieving health literacy, adopting health‐enhancing behaviors, and promoting the health of others. Comprehensive school health education includes curricula and instruction for students in pre‐K through grade 12 that address a variety of topics”	WSCC description;^1^ WellSAT 3.0 item NE1;^20^ HHFKA^10^
HE2. Specifies that health education is provided by qualified, trained professionals	“When provided by qualified, trained teachers, health education helps students acquire the knowledge, attitudes, and skills they need for making health‐promoting decisions, achieving health literacy, adopting health‐enhancing behaviors, and promoting the health of others.”	WSCC description^26^
HE3. Addresses health education for students in district	“Comprehensive school health education includes curricula and instruction for students in pre‐K through grade 12…”	Society of Health and Physical Educators (SHAPE) America^40^
HE4. Addresses alignment between health education curriculum goals and the needs of students in the community with the goal of reducing health inequity	“Health education, based on an assessment of student health needs and planned in collaboration with the community, ensures reinforcement of health messages that are relevant for students and meet community needs.”	SHAPE America^40^
HE5. Addresses National Health Education Standards (NHES)	“Health education curricula and instruction should address the National Health Education Standards (NHES)…”	WSCC description^26^
HE6. Incorporates the CDC's characteristics of an effective health education curriculum	“Health education curricula and instruction should… incorporate the characteristics of an effective health education curriculum.”	WSCC description^26^
HE7. Specifies that health education curriculum will be evaluated and revised	See HE6	SHAPE America^40^
HE8. Addresses opportunities for interdisciplinary connections and practicing health‐related skills outside of health education classes	“Students might also acquire health information through education that occurs as part of a patient visit with a school nurse, through posters or public service announcements, or through conversations with family and peers.”	SHAPE America^40^
HE9. Includes goals for nutrition education that are designed to promote student wellness in a manner that the local education agency determines is appropriate (Federal requirement)	“Comprehensive school health education includes curricula and instruction for students in pre‐K through grade 12 that address a variety of topics such as… healthy eating/nutrition”	WellSAT 3.0 item NE1;^20^ HHFKA^10^
Employee Wellness		
EW1. Designates employee wellness as a priority in the district organization structure	“A comprehensive school employee wellness approach is a coordinated set of programs, policies, benefits, and environmental supports designed to address multiple risk factors (eg, lack of physical activity, tobacco use) and health conditions (eg, diabetes, depression) to meet the health and safety needs of all employees.”	Healthy Workforce 2010;^41^ National Association of Chronic Disease Directors (NACDD)^42^
EW2. Includes dissemination of health education materials focused on skill development and lifestyle behavior change for school employees	“Partnerships between school districts and their health insurance providers can help offer resources”	Healthy Workforce 2010^41^
EW3. Addresses coordination with health insurance providers to conduct health risk screening	“Partnerships between school districts and their health insurance providers can help offer resources, including personalized health assessments…”	Healthy Workforce 2010;^41^ NACDD^42^
EW4. Addresses creating an environment that supports employees' healthy lifestyles	“Schools can create work environments that support healthy eating, adopt active lifestyles, be tobacco free, manage stress, and avoid injury and exposure to hazards (eg, mold, asbestos).”	Healthy Workforce 2010;^41^ NACDD^42^
EW5. Addresses social and emotional supports for school employees including the use of Employee Assistance Programs or other programs	“Fostering school employees' physical and mental health protects school staff, and by doing so, helps to support students' health and academic success.”	WSCC description;^1^ NACDD^42^
EW6. Includes use of employee input in design and evaluation of employee wellness programs	“A comprehensive school employee wellness approach is a coordinated set of programs, policies, benefits, and environmental supports designed to address multiple risk factors… and health conditions… to meet the health and safety needs of all employees”	Centers for Disease Control;^43^ NACDD^42^
EW7. Address tobacco use by school employees	See EW4	WSCC description;^26^ NACDD^42^
EW8. Addresses school employee adoption and modeling of healthy lifestyles	See EW4	WellSAT 3.0 WPM1^20^
EW9. Addresses promotion of a positive workplace climate	“Fostering school employees' physical and mental health protects school staff, and by doing so, helps to support students' health and academic success”	Healthy Workforce 2010^41^
EW10. Addresses space and break time for lactation/breast feeding	See EW1	Expert review
EW11. Addresses methods to communicate information about and encourage participation in available wellness programs	See EW1	Expert review; NACDD^42^
Health Services		
HS1. Addresses presence of qualified health service providers in district schools	“Qualified professionals such as school nurses, nurse practitioners, dentists, health educators, physicians, physician assistants and allied health personnel provide these services.”	American Academy of Pediatrics (AAP),^44^ National Association of School Nurses (NASN)^45^
HS2. Addresses community‐based service coordination and communication with providers to meet student health needs	“These services are designed to ensure access and/or referrals to the medical home or private healthcare provider.”	WSCC description;^26^ CDC School Health Services Model;^46^ NASN^45^
HS3. Addresses alignment of health services with the health needs of students in the community	“School health services actively collaborate with school and community support services to increase the ability of students and families to adapt to health and social stressors, such as chronic health conditions or social and economic barriers to health”	WSCC description;^1^ NASN^45^
HS4. Addresses engagement of and communication with families to address individual student health needs	“Health services connect school staff, students, families, community and healthcare providers to promote the healthcare of students and a healthy and safe school environment”	WSCC description;^26^ CDC School Health Services Model^46^
HS5. Specifies opportunities for dissemination of health information resources to students and families (eg, pamphlets, flyers, posters)	“…student and parent education complement the provision of care coordination services”	WSCC description;^1^ NASN^45^
HS6. Addresses student physical health screenings (eg, hearing, vision)	“…wellness promotion, preventive services and staff… complement the provision of care coordination services.”	NASN^45^
HS7. Addresses assessment and planning for chronic disease management to meet individual student needs (eg, asthma, diabetes, etc.)	“School health services intervene with actual and potential health problems, including …emergency care and assessment and planning for the management of chronic conditions (such as asthma and diabetes)”	WSCC description;^1^ CDC School Health Services Model;^46^ NASN^45^
HS8. Addresses management of allergies in the school environment	“School health services intervene with actual and potential health problems, including …emergency care and assessment and planning for the management of chronic conditions (such as asthma and diabetes)”	AAP^47^
HS9. Addresses provision of acute and emergency care	“School health services intervene with actual and potential health problems, including providing first aid…”	WSCC description;^1^ CDC School Health Services Model^46^
HS10. Specifies a health services plan for response to student sexual risk behavior (eg, HIV/STD, pregnancy)	“School health services intervene with actual and potential health problems”	AAP^44^
HS11. Specifies a health services plan for response to student substance use (eg, tobacco, alcohol, illicit substances)	See HS10	AAP^48^
Integration, Implementation, and Evaluation		
IIE1. Specifies use of Centers for Disease Control and Prevention's WSCC model or other coordinated/ comprehensive method to guide wellness activities	NA	WSCC description;^26^ Comprehensive Coding System to Measure the Quality of School Wellness Policies item CP84^16^
IIE2. Addresses the establishment of an ongoing district wellness committee	NA	WellSAT 3.0 item IEC1;^20^ HHFKA^10^
IIE3. Addresses how families, students, representatives of the school food authority, teachers of physical education, school health professionals, the school board, school administrator, and the general public will participate in the development, implementation, and periodic review and update of the local wellness policy	NA	WellSAT 3.0 item IEC2; ^20^ HHFKA^10^
IIE4. Addresses diverse representation on district wellness committee outside of federal requirements to reflect WSCC domains such as: A. employee wellness B. physical environment, custodial services C. behavioral health (counseling, psychological, social services) D. health education E. health services F. nutrition and physical activity providers in the community)	NA	NACCD^49^
IIE5. Addresses the establishment of an ongoing school building level wellness committee (note: this may also be called a school health team, school health advisory committee, or school health council, or similar name)	NA	WellSAT 3.0 item IEC8^20^
IIE6. Addresses the assessment of district implementation of the local wellness policy at least once every 3 years (Federal Requirement)	NA	WellSAT 3.0 item IEC5;^20^ HHFKA^10^
IIE7. Identifies the position of the LEA or school official(s) responsible for the implementation and oversight of the local wellness policy to ensure each school's compliance (Federal Requirement)	NA	WellSAT 3.0 item IEC3;^20^ HHFKA^10^
IIE8. Addresses a plan to assess the impact of wellness policy on behavioral health and educational outcomes (eg, student and employee attendance, office discipline referrals, BMI screenings	NA	NACCD^49^
IIE9. Addresses making triennial assessment results available to the public	NA	WellSAT 3.0 item IEC6;^20^ HHFKA^10^
IIE10. Identifies funding support for wellness activities	NA	Comprehensive Coding System to Measure the Quality of School Wellness Policies item E95;^16^ NACCD^49^
IIE11. Addresses a plan for updating policy based on results of the triennial assessment (Federal Requirement)	NA	WellSAT 3.0 item IEC7;^16^ HHFKA^49^
IIE12. Addresses use of culturally inclusive practices in school wellness activities	NA	CDC Characteristics of Effective Health Education^50^
IIE13. Identifies professional learning opportunities for district employees to support wellness policy implementation	NA	CDC Characteristics of Effective Health Education^50^
